# Impact of a medically supervised safer injecting facility on drug dealing and other drug-related crime

**DOI:** 10.1186/1747-597X-1-13

**Published:** 2006-05-08

**Authors:** Evan Wood, Mark W Tyndall, Calvin Lai, Julio SG Montaner, Thomas Kerr

**Affiliations:** 1 British Columbia Centre for Excellence in HIV/AIDS, St. Paul's Hospital, 608 - 1081 Burrard Street, Vancouver BC V6Z 1Y6, Canada; 2 Department of Medicine, University of British Columbia, 3300 - 950 West 10th Avene, Vancouver BC V5Z 4E3, Canada

## Abstract

North America's first medically supervised safer injecting facility (SIF) recently opened in Vancouver, Canada. One of the concerns prior to the SIF's opening was that the facility might lead to a migration of drug activity and an increase in drug-related crime. Therefore, we examined crime rates in the neighborhood where the SIF is located in the year before versus the year after the SIF opened. No increases were seen with respect to drug trafficking (124 vs. 116) or assaults/robbery (174 vs. 180), although a decline in vehicle break-ins/vehicle theft was observed (302 vs. 227). The SIF was not associated with increased drug trafficking or crimes commonly linked to drug use.

## Introduction

Despite existing interventions [1,2], illicit injection drug use continues to fuel infectious disease and fatal overdose epidemics in many settings, and has prompted substantial community concern [3-5]. Public health programming aimed at reducing the harms of illicit drug use commonly face community and legal opposition due to concerns that these services may lead to increases in criminal activity in their vicinity [6].

In an effort to address longstanding epidemics of HIV and drug-related overdose, Vancouver opened North America's first medically supervised safer injection facility (SIF) on September 22, 2003. Consistent with most SIF [7], within the facility, injection drug users (IDU) can inject pre-obtained illicit drugs under the supervision of medical staff, and an addictions counsellor and nursing care are available on site [8]. A major concern prior to the opening of the SIF was that the facility would result in increased migration of IDU and drug dealers to the city's Downtown Eastside where the facility is located, and subsequently prompt increases in criminal activity [8-11]. To examine these concerns, the present study was therefore conducted to examine patterns of criminal activity (drug trafficking and other drug-related crime) in the city's Downtown Eastside since the facility opened.

## Methods

For the present analyses, we accessed Vancouver Police Department statistics regarding charges for drug trafficking (which is defined to include selling, administering, giving, transferring, transporting, sending, or delivering illicit drugs), assaults and robberies, and vehicle break-ins and vehicle theft in the neighborhoods broadly defined as the Downtown Eastside area (Downtown Eastside proper, Chinatown, Gastown, Victory Square, and Strathcona). These indicators were selected for several reasons. First, although a reduction in public drug use and publicly discarded syringes has been attributed to the opening of the SIF [12], the potential influx of drug dealers to sell drugs to the SIF's clientele has not been thoroughly investigated. Second, rates of assaults and robberies and vehicle break-ins and vehicle thefts were evaluated to assess the potential of an increase in drug-related crime, since these activities have been attributed to a concentrated illegal drug scene in the neighborhood [12]. The categories of assaults and robberies and vehicle break-ins and theft were combined in the source data file and it was not possible to separate these indicators.

We compared the monthly average number of charges for these activities in the Downtown Eastside between October 1, 2003 and September 30, 2004 (pre-SIF year) versus the monthly average during the period October 1, 2004 and September 30, 2005 (post-SIF year). Since there were a limited number of data points to compare trends between years, data were plotted on line graphs, and average annual levels for each of the three indicators (drug trafficking, assaults and robbery, and vehicle break-ins/theft) were compared using paired *t*-tests.

## Results

**Figure 1:**
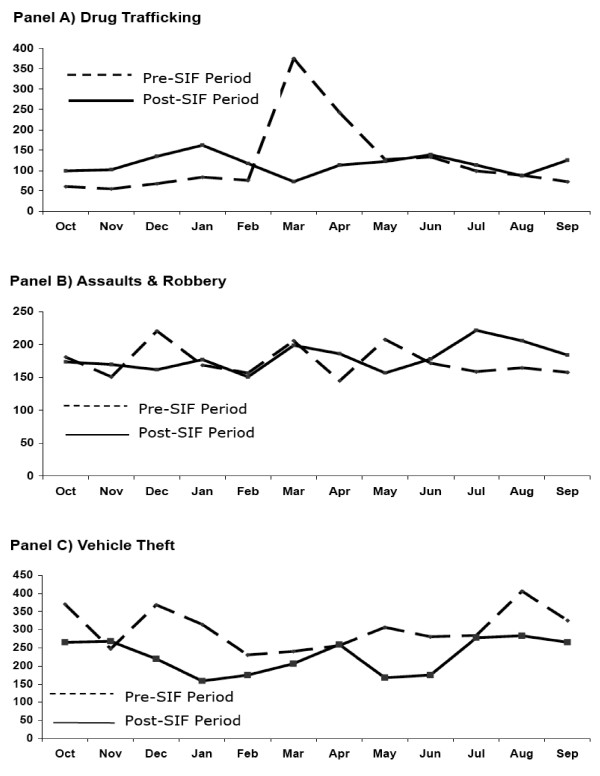
Monthly crude total number of charges for drug trafficking (Panel A), assaults and robbery (Panel B), and vehicle theft (Panel C) in the year before versus the year after the SIF opened.

The crude monthly totals for the year prior versus the year after the SIF opened are shown in Figure 1. As shown here, there were no obvious differences between the two years with respect to the various indicators of drug-related crime.

Similarly, using a *t*-test, no increases were seen with respect to: drug trafficking (124 [SD = 94] vs. 116 [SD 24]; mean difference = 7.9, *t*-stat = 0.26, df = 11, *p *= 0.803) and assaults/robbery (174 [SD = 25] vs. 180 [SD = 21]; mean difference = -6.2, *t*-stat = -0.59, df = 11, *p *= 0.565), although significant declines in vehicle break-ins/theft were observed (302 [SD = 57] vs. 227 [SD = 48]; mean difference = 75.7, *t*-stat = 4.22, df = 11, *p *= 0.001).

## Discussion

In the present study, when we compared annual periods before and after the opening of the Vancouver SIF, we found that rates of arrest for drug trafficking, assaults, and robbery were similar after the facility's opening, although rates of vehicle break-ins/theft declined significantly. These results are consistent with a recent study of the impact of Australia's first SIF which concluded that the Sydney facility was not associated with an increase in the proportion of drug use or supply offences [13].

The present study has several limitations. Most importantly, some crime statistics may be confounded by discretionary policing practices and levels of police deployment. Because of these issues, we did not investigate reports of drug possession, since the Vancouver Police Department has a discretionary approach towards drug possession. Specifically, in many instances police have directed or escorted IDU to the SIF, whereas during other periods an increased number of possession charges have been laid against IDU injecting in public reportedly as a police strategy to maximize use of the SIF and to reduce public disorder. Nevertheless, with respect to public drug use, an earlier study reported reductions in public drug use as measured by four independent measures [12]. In contrast to their approach to drug possession, the local police have a zero tolerance approach towards drug trafficking. Nevertheless, this measure suffers from uneven levels of police deployment between periods, which was evident in the drug trafficking data. Specifically, there are periodic increases in police activity that cannot be accounted for, and there was a spike in the drug trafficking data in the pre-SIF year coinciding with a well described police crackdown [14], and hence the small decline in drug trafficking charges in the post-SIF year is due to this effect. Finally, although the statistically significant decrease in vehicle break-ins/thefts in the post-SIF period does not appear to be due to the police crackdown in the pre-SIF period, due to the above concerns, we caution against inferring that this reduction was due to the SIF. Instead, we believe our overall findings suggest that the SIF was not associated with a marked increase in drug-related criminal activity.

In summary, the present study suggests that the opening of North America's first medically supervised safer injecting facility was not associated with a marked increase in drug trafficking or acquisitive crimes in the year after the facility opened. These findings suggest that the benefits of the SIF on public drug use and HIV risk behavior have not been offset by an increase in criminal activity in the neighborhood [12,15]. These findings should make a valuable contribution to the ongoing debates regarding the value of SIF, and for the cities in Canada and elsewhere that are considering initiating SIF trials [8-10,16-19].

## Competing interests

The author(s) declare that they have no competing interests.

## Authors' contributions

EW, CL, and TK designed the study. CL conducted the statistical analyses. EW wrote the first draft and compiled the co-authors' suggestions. All authors participated in the drafting of the manuscript and approved the final version.
